# Synergistic anti-methicillin-resistant *Staphylococcus aureus* (MRSA) activity and absolute stereochemistry of 7,8-dideoxygriseorhodin C

**DOI:** 10.1038/s41429-019-0275-8

**Published:** 2020-01-28

**Authors:** Bailey W. Miller, Joshua P. Torres, Jortan O. Tun, Malem S. Flores, Imelda Forteza, Gary Rosenberg, Margo G. Haygood, Eric W. Schmidt, Gisela P. Concepcion

**Affiliations:** 1 Department of Medicinal Chemistry, University of Utah, Salt Lake City, UT 84112 USA; 2 The Marine Science Institute, University of the Philippines Diliman, Quezon City, 1101 Philippines; 3 Academy of Natural Sciences Philadelphia, Drexel University, 1900 Benjamin Franklin, Parkway, PA 19103 USA

**Keywords:** Drug discovery and development, Small molecules

## Abstract

The emergence of antibiotic resistance necessitates not only the identification of new compounds with antimicrobial properties, but also new strategies and combination therapies to circumvent this growing problem. Here, we report synergistic activity against methicillin-resistant *Staphylococcus aureus* (MRSA) of the β-lactam antibiotic oxacillin combined with 7,8-dideoxygriseorhodin C in vitro. Ongoing efforts to identify antibiotics from marine mollusk-associated bacteria resulted in the isolation of 7,8-dideoxygriseorhodin C from a *Streptomyces* sp. strain cultivated from a marine gastropod tissue homogenate. Despite the long history of 7,8-dideoxygriseorhodin C in the literature, the absolute configuration has never been previously reported. A comparison of measured and calculated ECD spectra resolved the configuration of the spiroketal carbon C6, and 2D ROESY NMR spectroscopy established the absolute configuration as 6s,6aS. The compound is selective against Gram-positive bacteria including MRSA and Enterococcus faecium with an MIC range of 0.125–0.5 μg ml^−1^. Moreover, the compound synergizes with oxacillin against MRSA as observed in the antimicrobial microdilution and time-kill assays. Simultaneous treatment of the compound with oxacillin resulted in an approximately tenfold decrease in MIC with a combination index of <0.5, indicating synergistic anti-MRSA activity.

## Introduction

**Fig. 1:**
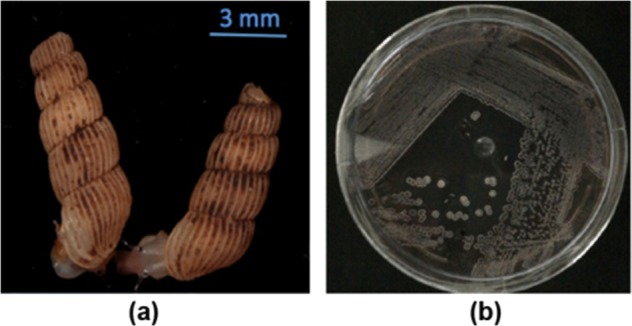
Animal and microbial sources of **1**. **a** *Truncatella guerinii* collected in Cebu, Philippines. **b** *Streptomyces* sp. (1425S.R.1a.1) grown on R2A agar with 2% NaCl isolated from the body tissue homogenate

Marine gastropod mollusks that are defended by thick shells harbor diverse culturable bacteria, many of which produce biologically active compounds [1–4]. Our continuing efforts to screen extracts from mollusk-associated bacteria against methicillin-resistant *Staphylococcus aureus* (MRSA) led us to the isolation of 7,8-dideoxygriseorhodin C (1) from *Streptomyces* sp. strain 1425S.R.1a.1. The bacterium was isolated from the body tissue homogenate of *Truncatella guerinii* (Fig. 1), a small gastropod that lives under plant debris near splash zones. Griseorhodins are aromatic polyketides known to be biologically active against Gram-positive bacteria [5–8]. In addition, some members of this structural family are known to be inhibitors of HIV reverse transcriptase and human telomerases [9]. The most recently isolated griseorhodins D, E, and F are intermediates and end products of post-PKS tailoring modification during biosynthesis [1].

Previously, *S. aureus* infections were primarily treated with β-lactam antibiotics, including oxacillin, a second-generation penicillin. Soon after the emergence of MRSA, these early generation β-lactam antibiotics were eliminated from the treatment options. To date, MRSA has become one of the most prevalent multi-drug resistant pathogens, and is responsible for most nosocomial and community-acquired infections worldwide [10, 11]. Currently, antibiotic treatments for MRSA infections are limited to glycopeptides (vancomycin and telavancin), oxazolidinones (linezolid and tedizolid), lipopeptides (daptomycin), and advanced-generation β-lactams, such as cephalosporins (ceftaroline).

Development of resistance to any antibiotic is inevitable. Apart from discovering new classes of antibiotics, combination drug therapy is another strategy to prevent or minimize the development of drug resistance [12]. Thus, we have begun to test compounds isolated from marine mollusk-associated bacteria that show anti-MRSA activity in combination with known antibiotics to identify potential synergistic interactions. When MRSA (ATCC® 43300™) was treated simultaneously with 7,8-dideoxygriseorhodin C and oxacillin, a more potent antimicrobial activity was observed compared with the single-drug treatments. This indicates possible synergistic activity, which may translate to a more rapid clearance of infection, shorter course of antibiotic therapy, and therefore, a reduction of dose-related toxicity [13].

Despite the long history of **1** in the literature, the absolute stereochemistry has never been reported. In addition, NMR chemical shifts were originally assigned without modern high-field NMR technology and advanced 2D experiments, and comparison with reported shift assignments for members of the structural family revealed inconsistently assigned signals. All carbon chemical shift signals were assigned, and the stereochemistry was determined to be 6*S*,6a*S* by using a combination of spectroscopic and computational methods.

## Materials and methods

### General experimental procedures

UV–Vis spectra were obtained by using a Shimadzu Prominence HPLC System (Shimadzu, Kyoto, Japan) coupled with a PDA detector. High-resolution electrospray ionization mass spectra were gathered using a QSTAR® XL Hybrid LC/MS/MS System (Applied Biosystems, Foster City, CA, USA) equipped with turbo ion spray source, delivering the sample at a rate of 40 µl min^−1^. NMR data were collected using a Varian 500-MHz NMR spectrometer with 5 mm Varian Oneprobe (^1^H 500 MHz, ^13^C 125 MHz). Residual signals from solvents were used for referencing. Semipreparative HPLC was performed using a Phenomenex C18 column (Luna 250 × 10 mm, 5 µm, 4.0 ml min^−1^). ECD spectra were obtained on an Aviv Biomedical Model 410 CD Spectrometer.

### Collection of animal material

*Truncatella guerinii* (specimen code PMS-1419Y) (Fig. 1) was collected in Tambuli East, Lapu-Lapu City, Cebu, Philippines in September 2009 and January 2011. Appropriate permits from the local government and the Bureau of Fish and Aquatic Resources (BFAR) were secured prior to sample collection. Two voucher specimens stored in 95% ethanol were prepared and deposited at the Marine Science Institute Museum and the Malacology Department of the Academy of Natural Sciences of Drexel University. Samples were immediately processed under sterile conditions in a temporary laboratory set-up at the collection site. A pool of five specimens was washed three times with sterile water before cracking the shells using a shell vise. Samples were dissected by Dr Alexander Fedosov of A.N. Severtzov Institute of Ecology and Evolution of the Russian Academy of Science. Individual tissues were extracted including the prostate gland, digestive tract, and the remainder of the body. Samples were placed in 1.5-ml microcentrifuge tubes and kept in an ice bath until microbial isolation.

### Isolation and identification of bacteria

After dissection, each tissue sample was homogenized in sterile sea water (1 ml) using mortar and pestle. The resulting homogenate was serially diluted (tenfold) and plated on R2A (Reasoner’s 2A) agar (0.2% yeast extract, 1% malt extract, 0.2% glucose) supplemented with 2% NaCl, 10 µg ml^−1^ nalidixic acid, 10 µg ml^−1^ cycloheximide, and 25 µg ml^−1^ nystatin. Plating for each dilution was done in duplicate inside a portable hood that was sterilized using UV and 70% ethanol. Primary or “mother” plates were incubated for 4 weeks at 30 °C. Bacteria were isolated, purified, and preserved in 20% glycerol at −80 °C. Bacteria with the code 1425S.R.1a.1 were isolated from the body of *T. guerinii* (specimen code PMS-1419Y). Genomic DNA of 1425S.R.1a.1. was extracted from a bacterial culture (7 ml) using the GenCatch™ Blood & Tissue Genomic Mini-Prep Kit (Epoch Life Science) following the manufacturer’s protocol. The 16S rRNA gene was amplified by polymerase chain reaction (PCR) using 20–50 ng template DNA, 6 µl of 2X PCR Master Mix (Promega), and 0.5 µM of primers: 27F (5′-AGAGTTTGATCCTGGCTCAG-3′) and 1492 R (5′-TACGGYTACCTTGTTACGACTT-3′), for a total reaction volume of 20 µl. Thermal cycling consisted of an initial denaturation step at 95 °C for 3 min, followed by 30 cycles of 95 °C for 30 s, 48 °C for 20 s, 72 °C for 1 min, and then a final extension step at 72 °C for 7 min. The amplicon was purified using the GenCatch™ Advanced PCR extraction kit (Epoch Life Science) and subsequently evaluated for correct size (~1400 bp) and homogeneity using 1% agarose gel electrophoresis before sending to Macrogen, Inc. (Seoul, South Korea) for gene sequencing. Two additional internal primers 518F (5′-CCAGCAGCCGCGGTAATACG-3′) and 800R (5′-TACCAGGGTATCTAATCC-3′) were used in sequencing. The four fragments were assembled and annotated using Geneious 8.1 software (Biomatters, New Zealand, available at http://www.geneious.com). Subsequently the contig was aligned against Ribosomal Database Project and GenBank nucleotide sequence databases using BLAST to identify similar bacterial 16S rRNA sequences. BLAST revealed that 1425S.R.1a.1 was 99% similar to *Streptomyces* sp. The 16S rDNA sequence was deposited in GenBank with accession code KY906645.

### Fermentation, extraction, and isolation

*Streptomyces* sp. (1425S.R.1a.1) was inoculated from a seed culture (100 ml) into eighteen 2.8 l-Fernbach flasks, each containing 1 l of R2A broth (0.2% yeast extract, 1% malt extract, 0.2% glucose, and supplemented with 2% NaCl), and cultured for 6 days at 30 °C with shaking at 150 rpm. The fermentation broth was centrifuged at 4000 rpm at 20 °C to separate the supernatant from the mycelia. The supernatant was extracted with HP20 diaion resin for 3 h. The diaion resin was recovered through filtration and washed with dH_2_O to remove salt before eluting the resin with 25, 50, 75, and 100% methanol (v/v). The methanol fraction was concentrated under reduced pressure and extracted with ethyl acetate. Finally, the ethyl acetate fraction was concentrated under reduced pressure to give the crude extract (620 mg). The crude extract was separated into six fractions on a C18 open column using a step gradient elution of acetonitrile and H_2_O with 0.1% TFA (20, 30, 40, 50, 60, 70, 80, 90, and 100%). Fraction 8 (90% acetonitrile (ACN)), which was active against MRSA, was subjected to silica gel column chromatography using (9.5:0.5) dichloromethane/methanol/0.1% acetic acid to yield bioactive fractions 9–3, 9–4, and 9–5 which were pooled (8.0 mg) and further purified by semipreparative HPLC (70% ACN: H_2_O + 0.05% TFA) to give 7,8-dideoxygriseorhodin C (1.7 mg).

7,8-dideoxygriseorhodin C (**1**) was obtained as a red powder, UV (MeOH + 0.01% TFA): λ_max_ 230, 256, 312, 352, 480, 505, 548 nm; ^1^H and ^13^C NMR (Table S1); HRESIMS *m/z* *=* 495.0889 [M + H]^+^ calculated for C_25_H_19_O_11_, 495.0927.

### Computational methods for ECD prediction

DFT calculations were performed at 298 K in methanol solution using the polarizable continuum model (PCM) in its integral equation formalism version (IEFPCM) incorporated into Gaussian 16 software. Models of all four diastereomers (6*S*,6a*S*;6*R*,6a*S*;6*R*,6a*R*;6*S*,6a*R*) were generated in Chem3D 15.0 (PerkinElmer). Each was energy minimized in Spartan (Wavefunction Inc.), and a conformer search was carried out at the molecular mechanics level of theory. Boltzmann distribution tables were generated for the top ten conformers of each diastereomer. The conformers that represented a combined > 99% of the population were geometry optimized at the b3lyp/6–31 g(d,p) level. TD-DFT calculations were performed at the b3lyp/6–31g(d′,p′) level with the number of states set to *N* = 40. Transitions were loaded into SpecDis 1.71 [14] for the generation of ECD spectra, and the spectrum from each conformer was weighted according to the Boltzmann distribution to produce a final averaged spectrum for each diastereomer.

### Computational methods for ^13^C NMR predictions

The top configurations of 6*S*,6a*S* and 6*R*,6a*S* were loaded into Gaussian 16. QM-NMR predictions were performed using the GIAO Method at the b3lyp/6–31g(d′,p′) level. Trimethylsilane (TMS) was modeled, optimized, and run with the same methods and at the same level of theory to be used as a reference.

### Determination of minimum inhibitory concentration (MIC)

The protocol was based on the Clinical Laboratory Standards Institute M7-A7 [15], with some modifications. Colonies of MRSA (ATCC® 43300™) were inoculated in 10 ml of cation-adjusted Meuller-Hinton broth (CAMHB) with 2% NaCl and incubated for 6 h, 37 °C, with shaking at 150 rpm. The turbidity of the bacterial suspension was adjusted with sterile broth to absorbance values of 0.08–0.1 at 625 nm to achieve a suspension containing ~1 × 10^8^ CFU ml^−1^. The adjusted inoculum was further diluted 100-fold and used for the assay. The inoculum was added into a 96-well flat-bottom microtiter plate containing 98 μl of CAMHB with 2% NaCl and 2 μl of each drug concentration. The final density of the bacteria per well was ~5 × 10^5^ CFU ml^−1^. Afterward, the plate was sealed and incubated with shaking for 18–24 h at 37 °C. Finally, 20 μl of 0.02% resazurin was added into each well and the fluorescence signals were measured at 530 nm excitation and 590 nm emission using a microplate reader (Biotek Synergy™ HT, VT, USA). MRSA treated with oxacillin and vehicle solvent was used as positive and growth controls, respectively. The concentration of DMSO per well did not exceed 1%. The results of the assays were expressed as % inhibition relative to the negative control using the following equation:

$$ {\% \ \rm{Inhibition}} ={\left( {1\,-\, \left( {\frac{{\rm{Absorbance}\,\rm{treated}\,-\,\rm{Absorbance}\,\rm{media}}}{\rm{Absorbance}\,\rm{vehicle}\,-\,\rm{Absorbance}\,\rm{media}}} \right)} \right)}\times\,100 $$

The data points were plotted in GraphPad Prism version 5 and inputted in CompuSyn software [16] for the determination of MIC. The MIC is defined as the lowest concentration of the antimicrobial agent that inhibits 97–100% of growth.

### Evaluation of synergistic activity

The synergistic activity of 7,8-dideoxygriseorhodin C with oxacillin was determined by performing the antimicrobial microdilution broth assay as described above. MRSA was treated with serial twofold concentrations of the compounds concurrently at constant 1X MIC ratio. The MIC of the individual drugs was also determined simultaneously as single-drug controls. To determine synergism or antagonism, the combination index (CI) at MIC_97_ was analyzed using CompuSyn [16] based on the equation below [17, 18]. CI ≤ 0.5, >0.5–4, >4 mean synergism, additive, antagonism, respectively [19].

$$ {{\rm{CI} = \frac{{\rm{MIC}_{\rm{a}}\,\rm{combination}}}{{\rm{MIC}_{\rm{a}}\,\rm{individual}}} + \frac{{\rm{MIC}_{\rm{b}}\,\rm{combination}}}{{\rm{MIC}_{\rm{b}}\,\rm{individual}}}}} $$

### Time-kill assay

Time-kill assays were done in 96-well microplates. MRSA (5 × 10^4^ CFU/well) was treated singly and in combination with **1** (0.0625 and 0.125 µg ml^−1^) and oxacillin (4 and 8 µg ml^−1^), and then 10 μl were aliquoted in each well at 2, 4, 8, 12, and 24 h post treatment, and serially diluted by tenfold. Ten microliters of each dilution were spotted on antibiotic-free CAMHBII agar plates. Colonies from the last dilution with 10–100 colonies were counted after 20–24 h of incubation, and the CFU ml^−1^ was calculated. Each experiment was conducted in triplicate. Synergy was defined as ≥2 log10 decrease in colony counts at 24 h with the combination, compared with the most active single-drug alone.

### MTT cell viability assay

Quantification of cell viability was based on the metabolism of MTT (3-(4,5-dimethyl-2-thiazolyl)−2,5-diphenyl-2H-tetrazolium bromide) by the live cells [20]. MDCK (Madin–Darby canine kidney cells) ATCC® CCL-34™ and AA8 (Chinese hamster ovarian cells) ATCC® CRL-1859™ were used as in vitro models for toxicity testing. Briefly, cells were seeded into a flat-bottom 96-well plate at a density of 2 × 10^4^ cells/well and incubated at 37 °C for 24 h. Subsequently, cells were treated with **1**, doxorubicin (positive control) or vehicle control (1% DMSO) and exposed for 72 h. Afterward, the contents of the wells were discarded by sharp flicking the plate. Then, 15 μl of filtered MTT (5 mg ml^−1^ in 1X PBS) was added to each well and incubated at 37 °C, 5% CO_2_ for another 3 h. Finally, the formazan crystals formed from the reaction were solubilized with 100 μl of DMSO per well. After 5 min of shaking, the absorbance was read in a microplate reader (Biotek®, Synergy-HT, VT, USA) at 570 nm. The results of the assays were expressed as percent cell viability relative to the negative controls using the following equation:

$$ {{\% \ \rm{Cell}\,\rm{viability} = \left( {\frac{{\rm{Absorbance}\,\rm{treated} - \rm{Absorbance}\,\rm{media}}}{{\rm{Absorbance}\,\rm{vehicle} - \rm{Absorbance}\,\rm{media}}}} \right)\times\,100}} $$

## Results and discussion

### Isolation, structure verification, and chemical shift reassignment

A *Streptomyces* sp. bacterium was isolated from the body tissue homogenate of a marine mollusk, *T. guerinii* obtained from Cebu, Philippines. The bacterium was grown in 18 l of R2A media supplemented with 2% NaCl, the cells were removed by centrifugation, and the culture medium was extracted with hydrophobic resin. The resin was washed, and then eluted with MeOH to produce a dark red crude extract that showed antibacterial activity against methicillin-resistant *S. aureus* (MRSA, ATCC® 43300™). Assay-guided purification of active constituents provided a series of red pigments, most of which appeared to be known analogs of the griseorhodin family based on spectroscopic data.

**Fig. 2:**
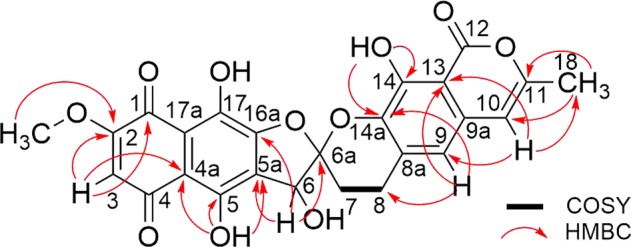
Key NMR correlations for the structural assignment of **1**

The most hydrophobic compound, based on retention in C18 reversed-phase chromatography, was isolated as a red solid. High-resolution mass spectrometry showed a protonated molecule at *m/z* = 495.0889 (M + H)^+^, suggesting a molecular formula of C_25_H_18_O_11_ with 17 degrees of unsaturation (Fig. S1), consistent with the formula of 7,8-dideoxygrisheorhodin C (**1**). Furthermore, the 1D ^13^C NMR experiment and UV–Vis absorption spectra matched the reported values for 7,8-dideoxygrisheorhodin C [21]. The ^1^H NMR spectrum, however, revealed slight changes in the chemical shifts of the two pairs of methylene protons on carbons 7 and 8 (Table S1). 2D NMR experiments, including gCOSY, gHSQCAD, and gHMBCAD, were used to verify the planar structure to be identical to previous reports (Fig. 2).

As previously stated, the ^13^C NMR spectrum was identical to reports in the literature. However, during the structure verification it became clear that there were significant misassignments of chemical shifts, particularly within the proton-deficient polycyclic regions. This is likely due to the original reports relying solely on 1D ^1^H and ^13^C NMR data, without the aid of modern 2D experiments.

The original report lists the ^13^C and ^1^H shifts at C7 as 21.30 and 1.99/1.92 ppm, while C8 was *δ* 23.24 and 2.51/2.43 ppm. However, it is clear from the HSQC data that the more upfield proton set (*δ* 2.16/2.40) should be associated with the carbon signal at *δ* 23.6 ppm, while the methylene protons at *δ* 3.05 are on the carbon at *δ* 21.7 ppm (Fig. S4). An HMBC correlation from H9 (*δ* 6.86) to the carbon at *δ* 21.7 supports this being C8, while C7 is at *δ* 23.6. Furthermore, the different chemical shifts for the protons on C7 support this being adjacent to the C6a stereocenter, while the protons on C8 are seen as a single peak with an integration of 2. These results mirror those of the structurally related compound hyaluromycin, in which the proton chemical shifts at C7 are also more upfield in comparison with those at C8 [22]. The chemical shifts of the nonprotonated carbons C1, C2, C4, C4a, C5, C12, and C17 were confirmed, while carbons C5a, C6a, C11, C13, C14, C14a, and C16a were all reassigned, based on HMBC correlations (Fig. 2).

In addition, six carbon nuclei (C4, C8a, C9a, C12, C17, and C17a) remained with no 2D NMR correlations to validate chemical shift assignment. Thus, QM-DFT calculations were run in Gaussian 16 to predict the ^13^C NMR chemical shifts for all nuclei in the compound. The results increased confidence for the placements of the 20 carbons assigned based on 2D NMR data. The remaining six carbons were all able to be assigned based on their relative shift values and confirmed with comparison with the calculated results (Table 1).

**Table 1:** Carbon chemical shift reassignment in 7,8-dideoxygriseorhodin C

Position	(1)	Original [21]	Calculated
	*δ*_C_	*δ*_C_	*δ*_C_
1	180.5	179	184.2
2	160.7	160.1	161
2-OCH_3_	57.5	56.94	57.6
3	110.6	110.1	111.4
4	186.1	184.5	185.8
4a	106.9	106.3	109.6
5	157.5	158	154.8
**5a**	**124.5**	**130.2**	**126.8**
6	74.5	74.1	80.5
**6a**	**113.6**	**152.1**	**118.5**
**7**	**23.6**	**21.3**	**28.3**
**8**	**21.7**	**23.2**	**27.5**
*8a*	*132.9*	*124.2*	*134.3*
9	115.4	114.8	114.5
*9a*	*130.7*	*124.2*	*131.4*
10	104.2	103.7	107.1
**11**	**152.7**	**148.7**	**155**
12	166.1	165.6	166.6
**13**	**104.7**	**113.3**	**106.5**
**14**	**149.1**	**147.9**	**150.2**
**14a**	**138.2**	**130.2**	**137.8**
**16a**	**147.1**	**137.8**	**146.8**
17	154.1	154.1	153.9
17a	114.5	113.9	117.3
18	19.1	18.5	22.6

Shifts that were reassigned based on 2D NMR experiments are shown in bold, while shifts that did not have 2D NMR correlations, reassigned purely by comparison with calculated values are in italics

Taken together, these data verify that the isolated compound is the same planar structure as the originally reported 7,8-dideoxygriseorhodin C. Furthermore, a combination of experimental and computational methods corroborated the reassignment of chemical shift data to the planar structure of the molecule. However, there remains a discrepancy in the proton shifts for the two methylene groups between the original report of 7,8-dideoxygriseorhodin C and our report here. Because the original papers did not report any stereochemical analysis, it is possible that the discrepancy arises from a difference in configuration between the original isolation and the material reported herein.

### Absolute configuration of 7,8-dideoxygriseorhodin C

Two stereocenters are present in **1** at carbons 6 and 6a. Interestingly, previous reporting on the absolute configurations of griseorhodins A and C showed the configuration of carbon 6a to be *S* in both compounds, while carbon 6 is identified as *S* in griseorhodin A and *R* in griseorhodin C [23, 24]. Despite being known for many years, no reports have been made on the absolute or relative configuration of 7,8-dideoxygriseorhodin C.

**Fig. 3:**
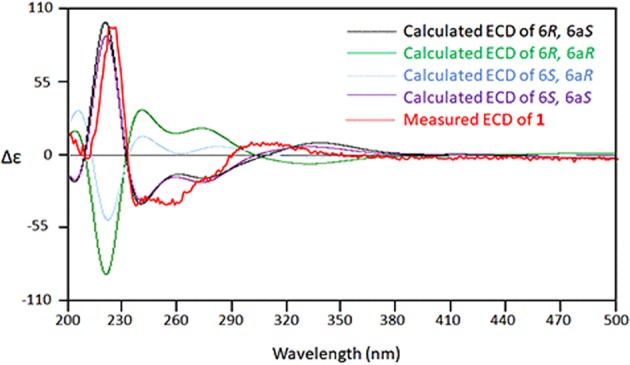
Experimentally derived (red) vs. calculated ECD spectra of each possible diastereomer of 7,8-dideoxygriseorhodin C. 6*R*,6a*S* (black) and 6*S*,6a*S* (purple) both match the experimental data, but are not distinguishable from one another

Stereochemical assignment was done through a combination of calculated vs. experimental ECD spectra and through-space NOE correlations. The four possible diastereomers (6*R*,6a*R*; 6*R*,6a*S*; 6*S*,6a*R*; 6*S*,6a*S*) were modeled and imported to Spartan to perform a conformer search. The free energy of each conformer was used to create a Boltzmann distribution, and those that represented a combined >99% of the population of each diastereomer were imported to Gaussian 16. These were geometry optimized, and TD-DFT calculations were performed to calculate transitions, which were imported into SpecDis 1.71 for the generation of calculated ECD spectra. Spectra were weighted according to the Boltzmann distribution and combined to create a single, averaged spectrum for each diastereomer, then compared with the experimental result (Fig. 3).

The stereocenter at carbon 6a has a dramatic effect on the calculated ECD spectrum, clearly supporting the assignment of carbon 6a as the *S* configuration, which is consistent with the previous reports within this structural class [23, 24]. The calculated spectra for 6*S*,6a*S* and 6*R*,6a*S*, however, are indistinguishable by this method, leaving the configuration of C6 ambiguous.

**Fig. 4:**
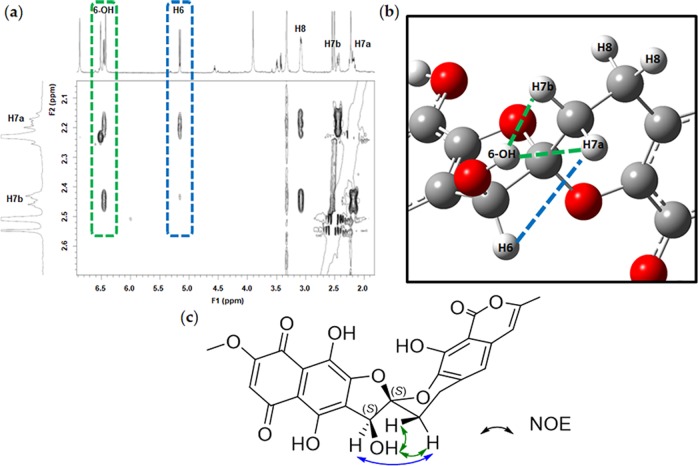
ROESY correlations for determination of absolute stereochemistry. **a** Spectrum centered on the relevant protons. Both H7a and H7b have clear ROESY cross-peaks with the hydroxyl proton 6-OH (green). These signals were in phase with cross-peaks between protons on C7 and C8, and out of phase with signals arising from exchangeable phenolic protons, indicating real ROESY correlations. Only one, however, shows a correlation to H6. **b** 3D spatial orientation of H6, 6-OH, H7a, and H7b from Gaussian generated model for 6S,6aS stereoconfiguration. In this orientation, the 6-OH is positioned close to and approximately equidistant from the two methylene protons on carbon 7, while H6 is oriented further from both, especially H7b. **c** Absolute configuration of **1** with supporting NOE correlations

Based on the 3D modeling, the configuration of C6 will have a significant effect on the spatial relationships between H6, 6-OH, and the diastereotopic protons H7a and H7b. In the model for 6*R*,6a*S*, H6 is oriented towards H7a and H7b within 2.8 Å of both, while 6-OH is pointed away. Alternatively, in 6*S*,6a*S*, the hydroxyl group is in the position close to the C7 protons, while H6 is 3.17 and 3.68 Å away from H7a and H7b, respectively (Fig. 4b). The ROESY spectrum shows a clear cross-peak between the C6 hydroxyl proton (*δ*_H_ 6.42) with both H7a and H7b (*δ*_H_ 2.16 and 2.40) (Fig. 4a). These cross-peaks are in the positive phase, while 6-OH cross-peaks with the phenolic protons are in the negative phase, indicating real NOE correlations. H6 (*δ*_H_ 5.12), however, has a weaker cross-peak with H7a, and almost no cross-peak with H7b. Taken together, these correlations can only be explained by the spatial orientation of the 6*S*,6a*S* absolute configuration (Fig. 4c).

### Synergistic anti-MRSA activity of 7,8-dideoxygriseorhodin C and oxacillin

The MICs of **1** against ESKAPE pathogens and other Gram-positive bacteria were evaluated using the microdilution broth assay. Compound **1** is active in all Gram-positive bacteria tested (*S. aureus*, MRSA, *Bacillus subtilis*, *Enterococcus faecium*, *Staphylococcus epidermidis*), but not in Gram-negative bacteria (Table S2). ATCC® 43300™ is an oxacillin- and methicillin-resistant strain that contains staphylococcal chromosome cassette *mec* (SCC*mec*) type II encoding the *mec* methicillin-resistance operon. **1** has in vitro efficacy when administered as a single agent with an MIC of 0.08–0.12 μg ml^−1^ (0.12–0.25 μM), which was much lower than oxacillin (1.59–6.24 μg ml^−1^ or 3.96–15.6 μM) (Table 2). Moreover, **1** showed virtually no cytotoxicity in MDCK and AA8 (Chinese hamster ovarian cells), with IC_50_ of 15.84 μg ml^−1^ (32 µM) and >49.5 μg ml^−1^ (100 µM), respectively (Fig. S7).

**Table 2:** Individual and combined effects of 7,8-dideoxygriseorhodin C (**1**) and oxacillin

Individual drugs	MIC against MRSA (μg ml^−1^)	Combination index at 1× MIC
**1**	0.08–0.12	
Oxacillin	1.59–6.24	
Combination at 1 × MIC ratio		
**1**	0.01–0.02	0.12–0.24
Oxacillin	0.02–0.298	

**Fig. 5:**
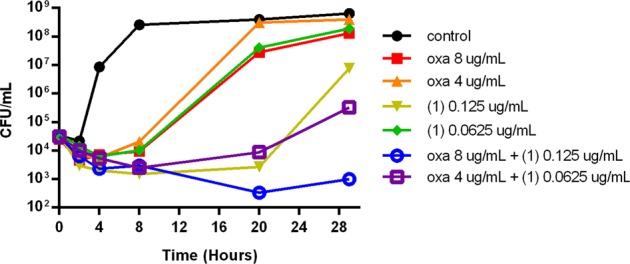
Synergistic anti-MRSA activity of 7,8-dideoxygriseorhodin C and oxacillin using the time-kill assay. MRSA was treated singly or in combination with subinhibitory concentrations of 7,8-dideoxygriseorhodin C (**1**) and oxacillin (oxa) and CFU/mL were determined at 0, 2, 4, 8, 20, and 30 h

One strategy to decrease toxicity and slow down the development of drug resistance is through combination therapy [13]. To investigate for possible synergistic activity, ATCC® 43300™ MRSA strain was treated simultaneously with **1** and oxacillin at 1 × MIC ratio. Table 2 shows the reduction of the individual MICs after combination treatment, indicating an enhanced antimicrobial activity compared with the single-drug treatments. To assess the synergistic activity, we used the CI method developed by Chou and Talalay [18]. For antimicrobial combinations, CI ≤ 0.5, >0.5–4, >4 indicate synergism, additivity, and antagonism, respectively. The CI for the combination of **1** and oxacillin at MIC ratio is 0.12–0.24, which indicates synergistic effects. This is consistent with the time-kill assay, which shows ≥100-fold decrease in CFU/mL between the combination and its most active constituent after 24 h (Fig. 5).

Our results show the potential renewed utility of an existing β-lactam antibiotic (oxacillin) in treatment of MRSA when used in synergy with **1**. Other existing β-lactam antibiotics that are already approved for human use are also currently being restored as treatment against MRSA. Three distinct generations of β-lactam antibiotics (meropenem, piperacillin, and tazobactam) were reported to be highly synergistic against 72 clinical strains of MRSA when used as a triple combination, with FICI (Fractional Inhibitory Concentration Index) of 0.11, while the double combinations of these drugs showed FICI of 0.22 (meropenem and piperacillin) and 0.44 (meropenem and tazobactam) against MRSA N315 (also with type II SCC*mec*) [25]. ND-421, an oxadiazole, was also found to work synergistically with oxacillin in vitro with FICI values of 0.31 and 0.37 against NRS70 and NRS123 MRSA strains, respectively, as well as in vivo in a mouse neutropenic thigh model [26]. The synergistic effect is proposed to be due to simultaneous perturbation of several components of cell wall synthesis in MRSA [25, 26]. In the oxadiazole-β-lactam combination, ND-421 inhibits PBP2a [27] while oxacillin inhibits PBP2 of MRSA [26]. The transpeptidase domain of PBP2a and transglycosylase domain of PBP2 are both required in cell wall biosynthesis of MRSA in the presence of β-lactam antibiotics [28].

In conclusion, the ongoing screening of mollusk-associated microbes led to the isolation of several known members of the griseorhodin structural class. One of these, 7,8-dideoxygriseorhodin C (**1**), demonstrated Gram-positive selective antimicrobial activity in the absence of mammalian cell toxicity. Excitingly, **1** also shows synergistic activity with the β-lactam antibiotic oxacillin, as demonstrated by the CI of 0.12–0.24. The absolute stereochemistry of **1**, which has never been previously reported, was found to be 6*S*,6a*S*. It is noteworthy that the stereoconfigurations of griseorhodins A and C have been solved, and in both cases the spiroketal carbon is also in the *S* configuration. However, carbon 6 was found to have the *S* configuration in griseorhodin A, and the *R* configuration in griseorhodin C. Finally, numerous inaccuracies in the literature regarding chemical shift assignments were rectified using 2D NMR experiments, as well as QM-DFT calculations.

## Supplementary information

Supplemental Material
