# Supplementary material for: Synergistic anti-methicillin-resistant Staphylococcus aureus (MRSA) activity and absolute stereochemistry of 7,8-dideoxygriseorhodin C
Source: J Antibiot (Tokyo). 2020 Jan 28;73(5):290–8. doi: 10.1038/s41429-019-0275-8 (PMC7125055; doi:10.1038/s41429-019-0275-8)
Supplement: Supplementary file 1 — Supplemental Material [file 41429_2019_275_MOESM1_ESM.docx]

*Supplementary Material*

Synergistic anti-methicillin-resistant *Staphylococcus aureus* (MRSA) activity and absolute stereochemistry of 7,8-dideoxygriseorhodin C

Bailey W. Miller ^1†^, Joshua P. Torres ^1,2†^, Jortan O. Tun ^2^, Malem S. Flores ^2^, Imelda Forteza ^2^, Gary Rosenberg ^3^, Margo G. Haygood ^1^, Eric W. Schmidt ^1^ and Gisela P. Concepcion ^2^*

^1^ Department of Medicinal Chemistry, University of Utah, Salt Lake City, UT 84112, United States of America

^2^ The Marine Science Institute, University of the Philippines Diliman, Quezon City 1101, Philippines

^3^ Academy of Natural Sciences Philadelphia, Drexel University, 1900 Benjamin Franklin Parkway, PA 19103, United Sates of America

^†^ These authors contributed equally to this work.

***** Correspondence: gpconcepcion@up.edu.ph; Tel.: +632-922-39-59


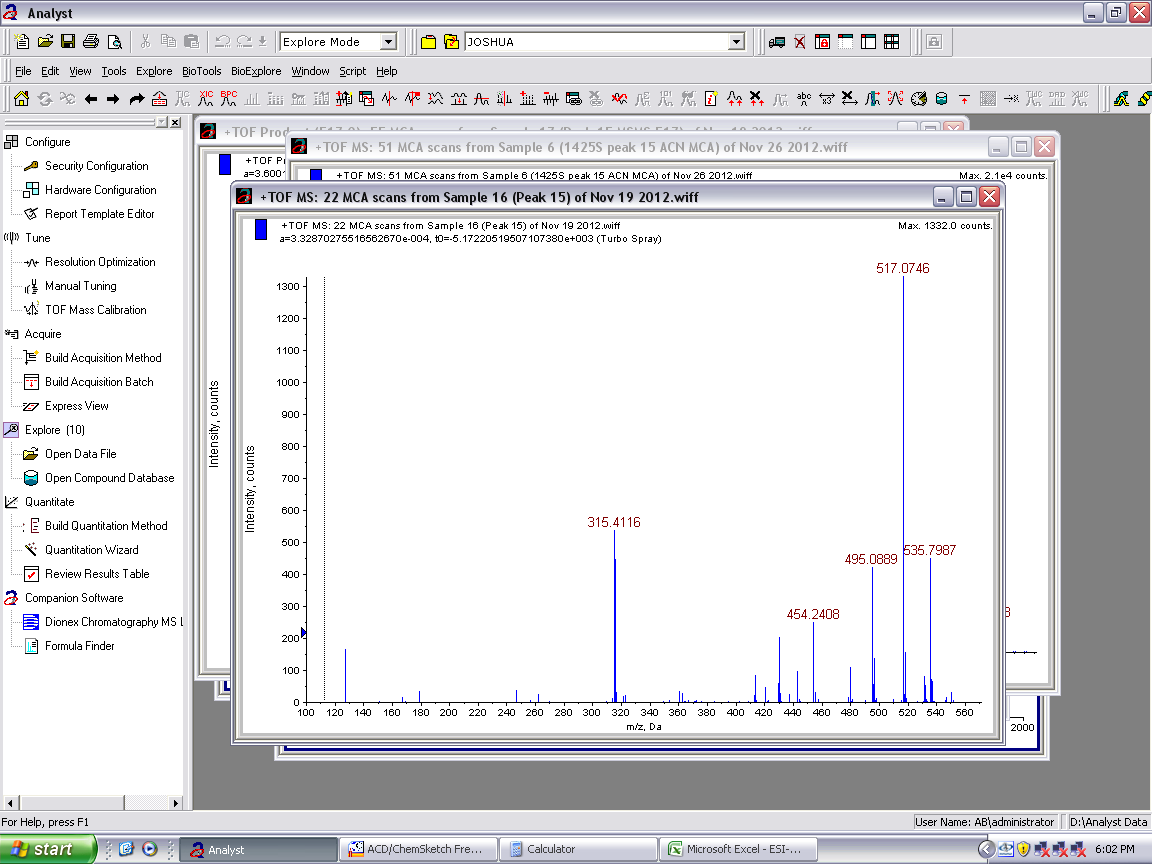


**Figure S1.** +ESI mass spectra of 7,8-dideoxygriseorhodin C

**Figure S2.** ^1^HNMR spectrum of 7,8-dideoxygriseorhodin C in DMSO-*d_6_*

**Figure S3.** ^13^CNMR spectrum of 7,8-dideoxygriseorhodin C in DMSO-*d_6_*

**Figure S4.** HSQC spectrum of 7,8-dideoxygriseorhodin C in DMSO-*d_6_*


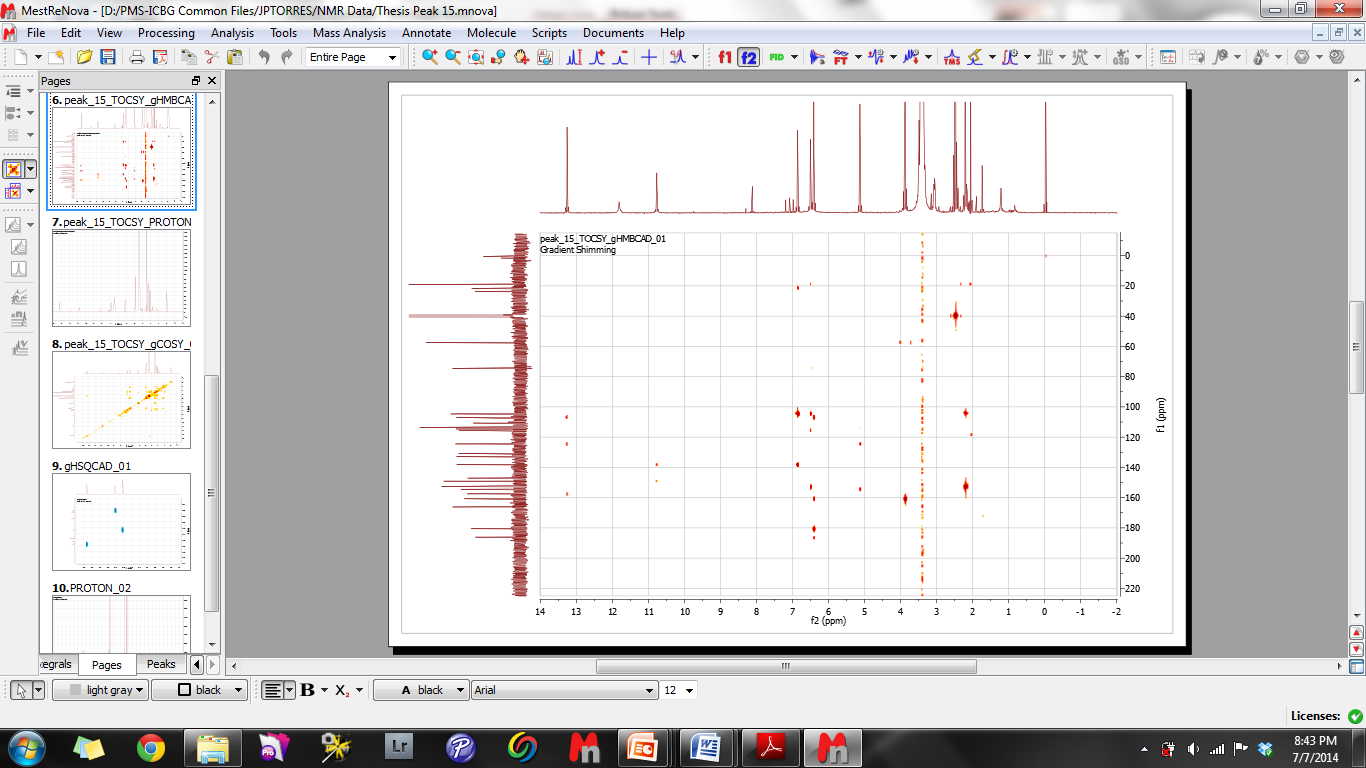


**Figure S5.** HMBC spectrum of 7,8-dideoxygriseorhodin C in DMSO-*d_6_*

**Figure S6.** ^1^H-^1^H COSY spectrum of 7,8-dideoxygriseorhodin C in DMSO-*d_6_*

**
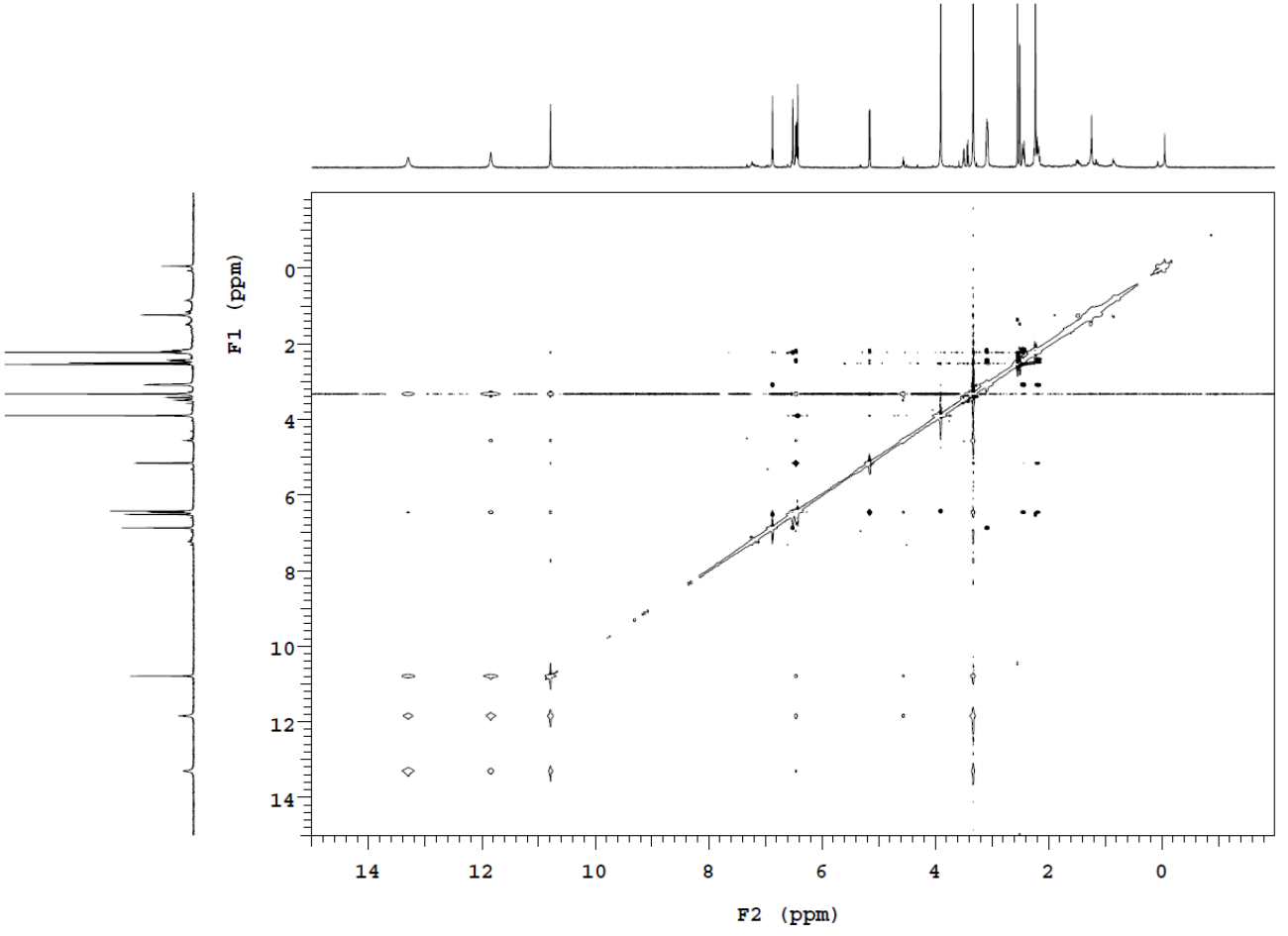
**

**Figure S7.** ROESY spectrum of 7,8-dideoxygriseorhodin C in DMSO-*d_6_*_. ­­_Filled cross peaks are in the positive phase, while empty (white) cross peaks are in the negative phase.


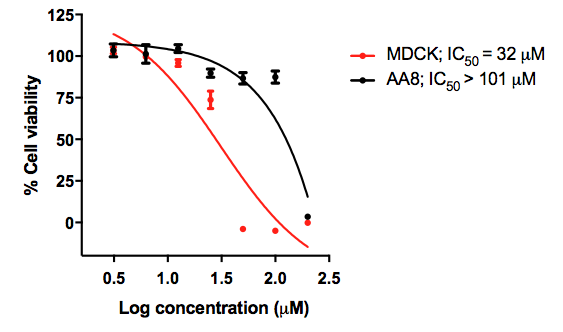


**Figure S8.** Cytotoxicity of 7,8-dideoxygriseorhodin C against MDCK and AA8

**Table S1**. ^1^H, ^13^C, COSY, HMBC data for compound **1** in DMSO-*d_6_*

| **C** | ***δ*_C_ (ppm)** | ***δ*_H_**  **(ppm, mult, *J* in Hz)** | **COSY**  **Correlations** | **HMBC**  **Correlations** |
| --- | --- | --- | --- | --- |
| 1 | 180.5 | - |  | - |
| 2 | 160.7 | - |  | - |
| 2-OCH_3_ | 57.5 | 3.87, 3H, s |  | 2 |
| 3 | 110.6 | 6.40, 1H, s |  | 1, 2, 5a |
| 4 | 186.1 | - |  | - |
| 4a | 106.9 | - |  | - |
| 5 | 157.5 | - |  | - |
| 5a | 124.5 | - |  | - |
| 5-OH | - | 13.26, 1H, s |  | 4a, 11, 5a |
| 6 | 74.5 | 5.12, 1H, s |  | 5a, 17, 6a |
| 6a | 113.6 | - |  | - |
| 6-OH | - | n.d. |  | - |
| 7 | 23.6 | 2.40, 1H, m  2.16, 1H, m | 8H  8H | - |
| 8 | 21.7 | 3.05, 2H, m | 7H | - |
| 8a | 132.9 | - |  | - |
| 9 | 115.4 | 6.86, 1H, s |  | 8, 13, 14a |
| 9a | 130.7 | - |  | - |
| 10 | 104.2 | 6.50, 1H, s | 18-H | 13, 11, 18 |
| 11 | 152.7 | - |  | - |
| 12 | 166.1 | - |  | - |
| 13 | 104.7 | - |  | - |
| 14 | 149.1 | - |  | - |
| 14a | 138.2 | - |  | - |
| 14-OH | - | 10.77, 1H, s |  | 14a, 14 |
| 16a | 147.1 | - |  | - |
| 17 | 154.1 | - |  | - |
| 17a | 114.5 | - |  | - |
| 17-OH | - | 11.81, 1H, bs |  | - |
| 18 | 19.1 | 2.20, 3H, s | 10H | 11, 13 |

**Table S2.** Minimum Inhibitor Concentrations (MICs) of 7,8-dideoxygriseorhodin C (**1**) against a panel of pathogens

| **Strains** | **MIC (*μ*g/mL)** | |
| --- | --- | --- |
|  | **1** | oxacillin |
| *Staphylococcus aureus* (ATCC^®^ 12600^™^) | 0.125 | 1 |
| methicillin-resistant *S. aureus* (ATCC^®^ 43300^™^) | 0.125-0.25 | 16 |
| *Staphylococcus epidermidis* (ATCC^®^ 35984^™^) | 0.125 | 2 |
| *Bacillus subtilis* (ATCC^®^ 6051^™^) | 0.125 | 1 |
| *Enterococcus faecium* (ATCC^®^ BAA-6569^™^) | 0.5 | >32 |
| *Klebsiella pneumoniae* (ATCC^®^ 13883^™^) | >16 | nt |
| *Enterobacter aerogenes* (ATCC^®^ BAA-35029^™^) | >16 | >32 |
| *Acinetobacter baumanii* (ATCC^®^ BAA-19606^™^) | >16 | >32 |
| *Mycobacterium tuberculosis* H37Ra (ATCC^®^ 25177^™^) | >16 | nt |
| nt = not tested |  |  |
